# Maternal–Fetal Infectious Risk and Early Antibiotic Treatment Patterns in Late Preterm and Term Newborns in a Romanian Secondary-Care Maternity: A Six-Month Observational Cohort

**DOI:** 10.3390/biomedicines14030538

**Published:** 2026-02-27

**Authors:** Anca Vulcănescu, Cătălina Elena Bica, Mirela-Anișoara Siminel, Sorin-Nicolae Dinescu, Maria-Magdalena Manolea, Sidonia-Maria Săndulescu, Virginia Maria Rădulescu, Valeriu Gheorman, Anda-Lorena Dijmărescu

**Affiliations:** 1 “Filantropia” Clinical Municipal Hospital, 200143 Craiova, Romania; 2 University of Medicine and Pharmacy of Craiova, 200349 Craiova, Romania; 3 Department of Pediatrics, University of Medicine and Pharmacy of Craiova, 200349 Craiova, Romania; 4 Department of Neonatology, University of Medicine and Pharmacy of Craiova, 200349 Craiova, Romania; 5 Department of Epidemiology, University of Medicine and Pharmacy of Craiova, 200349 Craiova, Romania; 6 Clinical Emergency County Hospital, 200642 Craiova, Romania; 7 Department of Obstetrics and Gynecology, University of Medicine and Pharmacy of Craiova, 200349 Craiova, Romania; 8 Department of Medical Informatics and Biostatistics, University of Medicine and Pharmacy of Craiova, 200349 Craiova, Romania; 9 Department of Obstetrics and Gynecology, Buna Vestire Clinic, 200345 Craiova, Romania

**Keywords:** suspected maternal–fetal infection, early-onset neonatal infection, maternal risk factors, antibiotic stewardship, neonatal biomarkers, Romania

## Abstract

**Background:** Microbiological confirmation of early-onset neonatal infection is inconsistently available in many secondary-care maternity units, and early management often relies on maternal risk profiles and inflammatory markers. **Objectives:** This study aimed to describe maternal infectious risk factors, neonatal biomarkers, and early antibiotic treatment patterns among late preterm and term newborns evaluated for suspected maternal–fetal infection (MFI) in a Romanian secondary-care maternity, and to explore whether a simplified bedside indicator (NeoSIR) corresponded with antibiotic initiation and treatment-related outcomes. **Methods:** Observational cohort study (April–September 2024) including newborns with gestational age ≥ 35 weeks evaluated within 24 h for suspected MFI based on maternal risk factors and/or neonatal clinical findings. We recorded maternal infections and screening results, neonatal complete blood count and C-reactive protein (CRP) at 24 h, cultures obtained as part of routine care, antibiotic initiation, treatment duration, and neonatal intensive care unit (NICU) admission. **Results:** Among 443 newborns, empirical antibiotics were initiated in 414 (93.5%) and 62 (14.0%) required NICU admission. Maternal urinary tract infection (UTI) documented during pregnancy (recorded diagnosis and/or positive urine culture and/or treatment) was associated with antibiotic initiation (*p* = 0.005). Elevated CRP (>0.5 mg/dL) was associated with antibiotic selection (*p* = 0.032) and longer therapy (mean 8.33 vs. 6.98 days, *p* < 0.001); courses were also longer in NICU-admitted infants (mean 10.39 vs. 6.81 days, *p* < 0.001). The exploratory two-item NeoSIR indicator (maternal UTI and CRP > 0.5 mg/dL) was feasible to compute but showed limited discrimination for antibiotic initiation in this high-treatment cohort. **Conclusions:** In this selected high-risk cohort, antibiotic exposure was highly prevalent and closely associated with maternal urinary tract infection history and neonatal CRP evolution. These findings primarily describe early management patterns in a secondary-care maternity with limited microbiological confirmation and should be interpreted as hypothesis-generating. The exploratory NeoSIR indicator is not intended as a diagnostic or decision-support tool and requires validation against culture-confirmed outcomes in more heterogeneous populations.

## 1. Introduction

Early-onset neonatal sepsis (EOS), defined as a bloodstream infection within the first 72 h of life, continues to be a major contributor to neonatal morbidity and mortality worldwide [1,2]. In high-income countries, routine maternal screening and intrapartum antibiotic prophylaxis have markedly decreased the incidence of Group B Streptococcus (GBS)-related EOS. However, infections occur, particularly among late preterm and term infants, where early signs can be subtle, diagnosis remaining complex, with severe outcomes [3,4]. Early recognition and intervention are critical, as delaying treatment increases the risk of complications and death [5].

EOS most often results from vertical transmission of pathogens during labor and delivery. GBS and *Escherichia coli* (*E. coli*) are the leading pathogens, accounting for the majority of cases in near-term and term infants [6,7]. While GBS remains dominant in many settings, *E. coli* has gained increasing importance, particularly in association with maternal urinary tract infections, intrapartum fever, or prolonged rupture of membranes. Other pathogens such as *Klebsiella pneumoniae*, *Staphylococcus aureus* (including methicillin-resistant strains), and *Candida* species, appear less frequently but can cause severe infections, especially in newborns requiring intensive care [6,8,9,10].

Current recommendations support early initiation of empirical broad-spectrum antibiotics, usually a beta-lactam combined with an aminoglycoside, to cover the most common pathogens [3,11]. However, the diagnosis of EOS remains challenging. Early signs, such as respiratory distress, feeding intolerance, or temperature instability are often nonspecific, and laboratory markers generally available like C-reactive protein (CRP) or complete blood count (CBC) lack sensitivity and specificity during the immediate postnatal period [12,13]. Procalcitonin has been proposed as a potentially more specific biomarker for early neonatal infection assessment in some settings; however, it was not routinely available in our secondary-care unit during the study period. Consequently, CRP remained the primary inflammatory biomarker used in bedside decision-making in this cohort. Blood cultures, while considered the diagnostic gold standard, suffer from low sensitivity due to small sample volumes, frequent maternal intrapartum antibiotic exposure, and, in some contexts, limited laboratory capacity, often failing to grow a pathogen despite the presence of infection [1,2,3].

As a consequence, many newborns are treated empirically despite negative cultures or the absence of true infection. Although this approach prioritizes safety, it exposes large numbers of infants to unnecessary antibiotics, which can disrupt the developing microbiome, foster antimicrobial resistance, and have long-term health implications [14]. These challenges are particularly evident in low-resource settings, including areas of Eastern Europe, where access to reliable microbiological testing is variable, and clinicians often rely on maternal history and simple biomarkers to guide treatment [15].

To reduce avoidable antibiotic use without compromising newborn safety, several multivariable risk assessment tools have been proposed. The most widely used, the Kaiser Permanente Neonatal EOS Calculator, combines maternal rick factors (fever, GBS status, duration of ruptured membranes) and neonatal parameters in order to generate individualized risk estimates [16,17]. Although such resources have helped reduce antibiotic use in high-resource environments, their implementation requires reliable maternal data and ample screening protocols, digital infrastructure, and local validation, which may not be feasible in many secondary-care settings. In such contexts, any simplified indicator should be interpreted cautiously and considered exploratory unless validated against robust clinical and microbiological endpoints.

Late preterm (35–36 weeks) and term infants (>37 weeks) represent a critical population in this context. Although their absolute risk of EOS is lower than that of very preterm neonates, they account for the majority of antibiotic exposure because of their large numbers and the overlap between early infection signs and normal postnatal adaptation [3,18]. In Romania and other Eastern European countries, secondary hospitals frequently function with limited diagnostic resources, underscoring the need for practical bedside tools which can be applied in any setting in order to identify infants at risk of infection [19].

In the EOS Calculator framework, neonatal status is categorized as well-appearing, equivocal, or clinically ill, based on objective bedside findings (e.g., respiratory distress/need for respiratory support, temperature instability, abnormal perfusion, altered responsiveness), which then modifies the management recommendation.

In this framework, maternal infection refers to clinically or microbiologically documented infectious conditions during pregnancy or labor; neonatal colonization reflects the presence of microorganisms on mucosal or skin surfaces without systemic illness; whereas true neonatal infection implies systemic inflammatory response with or without microbiological confirmation. In secondary-care settings where cultures are inconsistently available, these entities often overlap in clinical reasoning and may contribute to precautionary empirical antibiotic exposure.

In this context, the primary objective of this prospective observational cohort study was to characterize maternal infectious risk factors, neonatal biomarkers, and early antibiotic treatment pathways among newborns evaluated for suspected MFI in a secondary-care maternity setting. A secondary, exploratory objective was to assess whether a simplified bedside indicator (NeoSIR) corresponded with antibiotic initiation and treatment-related outcomes. NeoSIR was not intended as a validated diagnostic or decision-support tool but as a hypothesis-generating indicator reflecting real-world practice in settings with limited microbiological confirmation.

## 2. Material and Methods

### 2.1. Study Design and Setting

This was a prospective observational study conducted in a secondary-level maternity over a six-month period, between April and September 2024. All newborns were followed during their routine hospitalization, and no interventions outside standard care were performed. The primary purpose of the study was to evaluate maternal risk factors, neonatal biomarkers, and clinical outcomes in order to optimize antibiotic use in newborns at risk of infection.

### 2.2. Patients

Consecutive newborns with gestational age ≥35 weeks were eligible if they were evaluated within the first 24 h of life for suspected maternal–fetal infection (MFI), defined pragmatically as the presence of maternal infectious risk factors and/or neonatal clinical signs prompting laboratory testing and/or empirical antibiotic consideration. Neonatal clinical signs prompting infection evaluation included temperature instability, respiratory distress, feeding intolerance, tachycardia, lethargy, hypotonia, poor perfusion, or metabolic instability documented within the first 24 h of life. Maternal risk factors included documented urinary tract infection (UTI) during pregnancy, clinically abnormal vaginal discharge or positive genital cultures, prolonged rupture of membranes (>12 h), maternal intrapartum fever or suspected chorioamnionitis, meconium-stained or foul-smelling amniotic fluid, and incomplete prenatal surveillance compared to national recommendations (at least 8 prenatal visits with three key ultrasounds; blood typing and anemia assessment; screening for hepatitis B, HIV and syphilis; bacteriological screening including GBS testing; and screening for preeclampsia and gestational diabetes). Prenatal monitoring was classified as incomplete when one or more of the above benchmark elements were not documented in the maternal record.

Eligibility could be triggered by: (i) maternal infectious risk factors in the absence of neonatal symptoms, (ii) neonatal clinical signs suggestive of infection without documented maternal risk factors, or (iii) both. Neonatal clinical signs prompting evaluation included temperature instability, respiratory distress, feeding intolerance, tachycardia, lethargy, hypotonia, or metabolic instability recorded in the first 24 h.

When gestational age was recorded as an interval (e.g., 34/35), the upper bound was used for eligibility classification and descriptive reporting.

When Ballard-based gestational age was available, the higher of last-menstrual-period and Ballard estimates was used for eligibility classification, to minimize misclassification in records with incomplete obstetric dating.

Exclusion criteria were gestational age <35 weeks, stillbirths, newborns transferred immediately after delivery to tertiary centers, and infants born after uneventful pregnancies with complete recommended obstetric monitoring and no recorded infectious risk factors (low-risk population not targeted by this cohort).

### 2.3. Ethical Considerations

The protocol was approved by the Ethics Committee of the University of Medicine and Pharmacy of Craiova (approval no. 127/15 June 2023). Written informed consent was obtained from the mothers of all newborns enrolled.

### 2.4. Maternal and Neonatal Assessments

For mothers, routine infection screening and obstetric microbiology were performed according to local practice, and vaginal secretions and amniotic fluid samples were collected during labor or cesarean section when clinically indicated. For newborns, the standard early evaluation included gastric aspirate and peripheral swabs (umbilical, skin, and ear) obtained as part of routine care, alongside a complete blood count (CBC) and C-reactive protein (CRP) measurement at 24 h of life; CBC and CRP were repeated after 72 h when antibiotic therapy continued. Blood cultures (and cerebrospinal fluid analysis when clinically indicated) were performed at the treating clinician’s discretion but were not consistently available due to limited laboratory capacity. In this cohort, blood cultures were obtained in 20 newborns, with no microbiological confirmation. Culture results were therefore treated as descriptive supportive information rather than as a diagnostic reference standard. Given these constraints, culture results were treated as descriptive supportive information rather than as a diagnostic reference standard, and treatment decisions were primarily driven by maternal history, neonatal clinical presentation, and inflammatory markers. Where prenatal cultures were performed, the exact gestational age and interval to delivery were not consistently documented, so microbiological findings were treated as qualitative screening indicators rather than time-updated exposures.

### 2.5. Outcomes

The primary endpoint was prolonged antibiotic exposure, defined a priori as ≥10 antibiotic-days (days of therapy, DOT). We selected ≥10 antibiotic-days as a pragmatic threshold to distinguish short empirical courses (typically reassessed at 48–72 h) from extended treatment trajectories reflecting ongoing clinical concern, biomarker persistence, NICU-level monitoring, or escalation decisions. This operational cut-off was chosen to capture prolonged exposure relevant to antibiotic stewardship within the constraints of the available dataset. Secondary endpoints were (i) broad-spectrum escalation (use of any third-generation cephalosporin, carbapenem, fluoroquinolone, colistin, or linezolid during hospitalization) and (ii) neonatal intensive care unit (NICU) admission. Antibiotic initiation (yes/no) was reported descriptively as an early management indicator.

### 2.6. Antibiotic Management

Empirical antibiotic therapy was initiated according to institutional protocols when infection was suspected, (within the first 24 h) based on maternal history, neonatal clinical status, and age-specific laboratory thresholds (e.g., leukocytosis > 20 × 10^3^/µL, neutrophilia > 12 × 10^3^/µL, or CRP > 0.5 mg/dL at 24 h). First-line regimens were typically beta-lactam-based (e.g., ampicillin), with aminoglycosides or broader-spectrum agents added or substituted at the clinician’s discretion in the presence of clinical deterioration, rising inflammatory markers, or microbiological findings suggestive of pathogenic colonization. Clinical reassessment was routinely performed at 48–72 h to decide on continuation, narrowing, or discontinuation of therapy.

### 2.7. Data Handling

The variables collected included various maternal variables in order to track the maternal compliance to the national prenatal monitoring guidelines (infection history, bacteriological findings, number of checkups and ultrasounds), as well as multiple neonatal characteristics including but not limited to sex, gestational age, birth weight, NICU admission, and laboratory parameters (CRP levels, CBC results, bacteriological findings). Data were reviewed for duplicates and errors.

### 2.8. Statistical Analysis

All analyses were performed using SPSS version 26.0 (IBM Corp., Armonk, NY, USA). Categorical variables were compared using the Chi-squared test or Fisher’s exact test, as appropriate. Continuous variables were assessed for normality using the Shapiro–Wilk test; normally distributed data were analyzed with Student’s *t*-test (two groups) or one-way ANOVA (≥3 groups), while non-normally distributed data were compared using the Mann–Whitney U or Kruskal–Wallis test. Spearman’s rank correlation was used for monotonic associations between continuous variables. Results are reported as *n* (%) and as mean ± SD or median (IQR), as appropriate. Given the exploratory nature of this work, *p*-values are interpreted descriptively (two-sided α = 0.05).

Antibiotic exposure was summarized as antibiotic-days (i.e., the sum of days across all administered agents), which reflects total antimicrobial exposure when combination therapy is used. In addition to univariable comparisons, multivariable logistic regression models were fitted to identify independent predictors of (i) NICU admission and (ii) prolonged antibiotic exposure, defined a priori as ≥10 antibiotic-days (corresponding to ≥5 days of dual ampicillin-gentamicin therapy). Candidate covariates were selected for clinical relevance and data availability (gestational age, birth weight, sex, prolonged rupture of membranes >18 h, maternal UTI and cervical culture findings, neonatal resuscitation at birth, and early inflammatory markers including CRP, WBC, and platelet count). Continuous predictors were z-standardized; missing maternal microbiology results were handled using a missing-indicator approach. This was used because microbiology testing was not performed uniformly in routine care; thus, missingness was partly structural (‘not performed’) rather than random. A complete-case approach would have reduced the effective sample size and could introduce selection bias. Results are reported as adjusted odds ratios (aORs) with 95% confidence intervals. These regression models were constructed to estimate associations with antibiotic-related outcomes and NICU admission in this cohort and should not be interpreted as predictive or risk-stratification models. In parallel, length of therapy (LOT) was defined as the maximum number of days across administered agents, reflecting the duration of any antibiotic treatment regardless of combination use.

Covariate selection and model specification: For antibiotic-related endpoints (prolonged antibiotic exposure and broad-spectrum escalation), covariates were pre-specified a priori based on temporality and clinical plausibility: gestational age, birth weight, sex, rupture of membranes duration (>18 h), maternal urinary tract infection status, maternal screening cultures (cervical/vaginal and urine cultures; positive/negative; tests not performed coded using a missing-indicator), neonatal resuscitation at birth, and 24 h inflammatory markers (CRP2, WBC2, and PLT2). To minimize collinearity, Apgar-5 was not entered simultaneously with resuscitation; the primary models used resuscitation, with Apgar-5 explored in sensitivity analyses. Model 1 excluded NICU admission to avoid adjustment for a potential intermediate; Model 2 additionally included NICU admission as an early marker of illness severity and care intensity. Model 1 denotes the univariable (unadjusted) logistic regression model. Model 2 denotes the multivariable logistic regression model adjusted for predefined covariates, specified a priori based on clinical plausibility and data availability. Continuous predictors were modeled per 1 SD increase; rupture of membranes duration was log-transformed where skewness was substantial. Multicollinearity was assessed using variance inflation factors (VIF), and all retained predictors had VIF < 5. Because CRP at 24 h was measured selectively, the broad-spectrum escalation models used baseline laboratory values (CRP1, WBC1, PLT1) to maximize completeness.

### 2.9. Development of the NeoSIR Score

Because the Kaiser Permanente EOS calculator could not be applied consistently in this cohort (e.g., incomplete maternal GBS status and limited availability of key intrapartum parameters), we explored a simplified bedside indicator: the Neonatal Sepsis Inflammatory Risk (NeoSIR) score. Two binary components were specified a priori based on local availability and clinical relevance: elevated neonatal CRP (>0.5 mg/dL at 24 h) and documented maternal UTI during pregnancy. Each component contributed one point (range 0–2). Although leukocytosis and neutrophilia were recorded and examined in univariable analyses, neonatal leukocyte indices show substantial physiological variability during the first 24 h of life, which may limit their discriminative utility for early management decisions. Erythrocyte sedimentation rate (ESR) was not routinely used for immediate neonatal infection assessment in our unit and was not systematically available. NeoSIR was therefore evaluated as a secondary, exploratory indicator to contextualize early clinical management (antibiotic initiation and NICU admission) and should not be interpreted as a validated diagnostic or decision-support tool for culture-confirmed EOS.

### 2.10. Sample Size Considerations

The minimum required sample size was estimated using G*Power 3.1.9.7 (Heinrich Heine University Düsseldorf, Düsseldorf, Germany) for the χ^2^ test family (test of independence), assuming a two-sided significance level of α = 0.05, statistical power of (1 − β) = 0.95, and a medium effect size (Cohen’s w = 0.25). For comparisons involving three categorical levels (df = 2), the resulting minimum sample size was 248 participants. As this was an observational cohort with consecutive inclusion over a six-month period, all eligible newborns were included consecutively, resulting in an enrolled cohort of *n* = 443. This exceeds the estimated minimum and supports adequate power to detect at least medium associations in the main categorical analyses.

## 3. Results

### 3.1. Maternal Characteristics and Monitoring Practices

The study included 438 mothers, corresponding to the 443 newborns analyzed in this cohort. The mean maternal age was 27.6 ± 6.5 years (95% CI: 26.96–28.18), with a median of 27 years. Ages ranged from 14 to 50 years. The 5% trimmed mean (27.5 years) was close to the raw mean, suggesting a symmetrical distribution.

Maternal age was significantly associated with pregnancy monitoring (*p* < 0.001, Chi-square), but was not independently associated with neonatal antibiotic initiation. Women aged 20–34 years most frequently had complete antenatal monitoring (61.1%), followed by women ≥ 35 years (12.0%). Partial monitoring was reported in 13.1% of women aged 20–34 and in 1.8% ≥ 35 years. Non-monitoring was rare (0.7%) and observed only among mothers < 35 years. Spearman’s correlation confirmed a positive association between maternal age and compliance with antenatal care (r = 0.286, *p* < 0.05).

Gynecological visits followed the same trend (*p* < 0.001). In the 20–34 group, 38.5% attended more than nine prenatal visits, while only 1.4% of adolescents reached this level. Spearman’s correlation (r = 0.268, *p* < 0.05) confirmed the association. Ultrasound examinations also correlated with age (*p* < 0.001): 35.5% of women aged 20–34 had more than nine ultrasounds, compared with 7.2% of those ≥ 35 years and 0.5% of those < 20 years.

Compliance with infectious disease screening varied: TORCH testing was most frequent in the 20–34 age group (50.5% immunized), followed by those ≥ 35 (9.7%). Only one case of active toxoplasmosis was identified (20–34 group). Syphilis testing was generally high, though 10.9% of women aged 20–34 and 5.4% of adolescents were untested. HIV testing showed a similar pattern: highest in 20–34 years (64.0%), with non-testing more common in adolescents (5.7%) and younger adults (10.9%), but rare in ≥35 years (1.6%). TB screening showed the same distribution (r = 0.232, *p* < 0.05).

Hepatitis B and C testing was significantly associated with age (*p* < 0.001). Negative results were most common in the 20–34 group (63.1%). HBs antigen positivity was rare (0.7% in 20–34; 0.9% in ≥35). Anti-HCV antibodies were detected in one case (0.2%).

Urinary tract infections (UTIs) during pregnancy were significantly associated with age (*p* < 0.001). Positive urine cultures were most frequent in the 20–34 group (28.7%), followed by ≥35 years (5.7%) and <20 years (2.7%). Treatment rates mirrored prevalence (28.5% in 20–34; 5.7% ≥ 35). Correlation analysis did not confirm an association between maternal age and treatment initiation (r = 0.083, *p* > 0.05).

Cervical cultures were positive most often in the 20–34 group (26.9%). Treatment following positive culture was significantly associated with maternal age (*p* = 0.001; r = 0.157, *p* < 0.05).

At admission, pathological vaginal discharge was not significantly associated with age (*p* = 0.433; r = −0.030, *p* > 0.05). Vaginal cultures identified a variety of pathogens, including *Candida* spp., *E. coli*, *Klebsiella* spp., *Staphylococcus aureus*, and GBS, though more than half were sterile (*n* = 219). Amniotic fluid cultures were also sterile in most cases, with no significant variation by maternal age (*p* = 0.292). Urine cultures collected at admission were predominantly sterile, with no association between age and positivity (*p* = 0.373). The few positive findings (mostly *E. coli*) were clinically relevant and contributed to maternal infection diagnosis.

Duration of membrane rupture showed a significant association with maternal age (*p* = 0.001). Antibiotic initiation decisions were primarily associated with documented maternal infection and neonatal laboratory findings rather than maternal age. Intact membranes were documented in 54.1% of women aged 20–34. By contrast, the appearance of amniotic fluid did not differ significantly across groups (*p* = 0.926), with clear fluid predominating.

### 3.2. Neonatal Characteristics and Outcomes

During the six-month study period, 443 newborns met the predefined eligibility criteria and were included in the final analysis. The cohort had a near-equal sex distribution, with 218/443 (49.2%) females and 225/443 (50.8%) males. Birth weight analysis showed that most infants (83.5%, *n* = 369) were within the normal weight range. A smaller proportion were underweight (14.0%, *n* = 62), while only 1.8% (*n* = 8) had high birth weight.

When evaluating the relationship between maternal infectious status and neonatal surface or gastric colonization, no statistically significant association was identified. Specifically, maternal vaginal discharge at delivery did not correlate with positive neonatal gastric aspirate cultures (*p* = 0.77, Chi-square test). This finding suggests that isolated maternal vaginal colonization had limited predictive value for neonatal therapeutic decisions. In line with the study design, neonatal gastric aspirates and peripheral swabs were considered observational markers of colonization rather than diagnostic indicators of infection.

### 3.3. Statistical Associations Between Maternal Infectious Status, Neonatal Antibiotic Therapy, and NICU Admission

Analysis of maternal and neonatal infectious parameters revealed several significant associations with antibiotic initiation, as seen in Table 1. Elevated neonatal C-reactive protein (CRP > 0.5 mg/dL) was strongly associated with the start of antibiotic therapy (*p* = 0.032, Chi-square test). Similarly, a history of maternal urinary tract infection (UTI) during pregnancy significantly increased the likelihood of neonatal antibiotic treatment (*p* = 0.005).

In contrast, other maternal bacteriological findings (including positive cervical or vaginal cultures) did not show a significant association with antibiotic initiation (*p* > 0.95). Likewise, the presence of vaginal secretions at admission and the overall degree of pregnancy monitoring were not predictive of antibiotic therapy (*p* = 0.99 and *p* = 0.97, respectively).

These results indicate that while neonatal inflammatory markers (particularly CRP) and maternal UTIs were influential in guiding therapeutic decisions, maternal colonization findings and pregnancy monitoring alone did not significantly influence antibiotic initiation. Neonatal swabs and gastric aspirates were evaluated as observational indicators of colonization and did not show predictive value for clinical management.

Detailed analysis showed that among neonates with elevated CRP (≥0.5 mg/dL), 102/107 (95.3%) received ampicillin, and very few required broader-spectrum antibiotics (Table 2). The occasional use of ceftriaxone or imipenem–cilastatin reflects selective escalation in infants with clinical deterioration, rising inflammatory markers, or concern for resistant pathogens, rather than routine first-line neonatal therapy. These exposures highlight practice variability under uncertainty in settings with limited microbiological confirmation and reinforce the stewardship need for structured reassessment and escalation criteria.

### 3.4. Correlation of Maternal Infection with Antibiotic Initiation

Maternal urinary tract infection (UTI) during pregnancy remained one of the most influential maternal factors associated with neonatal antibiotic therapy (Table 3). A significant correlation was identified between documented maternal UTI and the initiation of antibiotics in newborns (*p* < 0.005). Clinicians consistently regarded confirmed maternal UTIs as a notable risk indicator, particularly in a setting where early neonatal diagnostics rely on simple laboratory markers.

Among the maternal urine cultures, *Escherichia coli* was the most frequently isolated organism, detected in 110 cases (24.9%). Of these, 102 newborns (92.7%) received early antibiotic treatment. Thirteen of the infants born to mothers with *E. coli* UTIs required NICU admission, representing 19.7% of all NICU admissions recorded during the study period. These figures illustrate the strong influence of maternal UTIs, especially those caused by *E. coli*, on clinical management immediately after birth.

Polymicrobial UTIs were less common but clinically relevant. Most involved *E. coli* in combination with *Enterococcus*, *Klebsiella*, *Staphylococcus*, or *Proteus* species. Despite their lower prevalence, these mixed infections were also followed by neonatal antibiotic initiation, underscoring that any maternal UTI with recognized pathogenic potential guided a cautious therapeutic approach.

Maternal vaginal secretion findings at admission and cervical culture results also contributed to the assessment of maternal infectious status; however, neither showed a statistically significant association with neonatal antibiotic initiation (*p* > 0.95) or with NICU admission (*p* > 0.90). Although microorganisms such as *Candida* spp., *E. coli*, *Klebsiella* spp., *Staphylococcus aureus*, and GBS were identified, these findings did not consistently alter neonatal therapeutic decisions. This pattern suggests that, compared with UTIs, lower-genital-tract infections were seen as less predictive of early neonatal complications in this cohort.

In addition, six mothers (1.4%) had documented SARS-CoV-2 infection during pregnancy. Two of these pregnancies also had positive cervical cultures, three had UTIs, and all six newborns received empirical antibiotics.

Neonatal inflammatory markers influenced treatment decisions in parallel with maternal risk factors. In addition to elevated CRP, increased total leukocyte count and high neutrophil proportion also contributed to the decision to initiate antibiotic therapy, even though these associations were not the strongest statistically. This reflects the practical reality of a resource-limited setting, where decisions rely mostly on accessible laboratory parameters, while highlighting the challenge of balancing early intervention with antibiotic stewardship in environments where diagnostic certainty is difficult to achieve.

### 3.5. Influence of Antenatal Monitoring on Neonatal Therapy

Antenatal monitoring varied widely among the mothers included in the study, yet these differences did not appear to meaningfully influence neonatal antibiotic initiation. Descriptive analysis showed that 71.9% of neonates born to mothers with complete prenatal monitoring received antibiotics, compared with 93.6% of those whose mothers had incomplete monitoring. Antibiotic initiation was most frequent among newborns from pregnancies with few or no documented prenatal visits or ultrasounds.

Despite these descriptive trends, statistical testing did not identify a significant association between the extent of antenatal monitoring and neonatal antibiotic therapy (*p* > 0.84 for all parameters). This indicates that, although incomplete monitoring was common among mothers whose newborns eventually received antibiotics, it did not independently predict treatment when considered alongside maternal infection and neonatal biomarkers.

These results highlight the distinct role of maternal infections (particularly UTIs) in shaping neonatal management. In contrast, the degree of prenatal monitoring did not influence whether antibiotics were started, despite the higher descriptive rates seen in incompletely monitored pregnancies. Clinicians appeared to base therapeutic decisions primarily on objective indicators present at birth, such as maternal infection status and neonatal laboratory abnormalities, rather than on the extent of maternal follow-up during pregnancy.

This approach reflects the practical realities in some areas, where immediate and accessible clinical information carries more weight than prenatal documentation when evaluating the risk of early neonatal infection.

### 3.6. Multivariable Associations with Antibiotic Exposure, NICU Admission, and Escalation

The length of antibiotic treatment (expressed as antibiotic-days) varied according to the newborns’ clinical and laboratory profiles. Elevated C-reactive protein (CRP > 0.5 mg/dL) at 24 h of life was associated with a longer treatment course, with infants in this group receiving antibiotics for an average of 8.33 days, compared with 6.98 days in those with normal CRP values (*p* < 0.001, Mann–Whitney U test). This difference reflects the strong influence of inflammatory markers on decisions to continue treatment beyond the initial empirical phase.

Among the 443 eligible newborns, empirical antibiotics were initiated in 414 (93.5%), and 62 (14.0%) required NICU admission. The most common initial regimen was ampicillin plus gentamicin (379/414, 91.5%). Total antimicrobial exposure, expressed as days of therapy (DOT; antibiotic-days), had a mean of 7.83 ± 2.87 and a median of 6 (IQR 6–10). The corresponding length of therapy (LOT) had a mean of 3.94 ± 1.35 and a median of 3 (IQR 3–5). Broad-spectrum escalation occurred in 81/414 infants (19.6%), including carbapenem exposure in 60/414 (14.5%). Compared with non-NICU infants, NICU-admitted newborns had longer DOT (median 10 (IQR 8–11.8) vs. 6 (IQR 6–10); *p* < 0.001) and longer LOT (median 5 (IQR 5–5) vs. 3 (IQR 3–5); *p* < 0.001) and were more likely to receive broad-spectrum agents (45.2% vs. 15.1%; *p* < 0.001).

Multivariable predictors of broad-spectrum escalation are presented in Table S1.

NICU admission was another factor associated with prolonged therapy. Newborns requiring intensive care received antibiotics for an average of 10.39 days, significantly longer than those managed on the postnatal ward (mean 6.81 days, *p* < 0.001). The extended duration likely reflects the need for closer monitoring and the greater clinical instability typically seen in infants admitted to NICU.

By contrast, the extent of maternal prenatal monitoring did not significantly affect antibiotic duration (*p* = 0.313). Whether mothers had comprehensive, partial, or absent antenatal care, treatment length in their newborns was determined mainly by neonatal findings, rather than by maternal follow-up history.

These patterns underscore the central role of early neonatal laboratory and clinical indicators in guiding treatment decisions. In a setting where definitive microbiological confirmation is often unavailable, clinicians relied on dynamic markers and the infant’s overall clinical course to judge the appropriate duration of therapy.

In multivariable analysis of prolonged antibiotic exposure (≥10 antibiotic-days), NICU admission remained the strongest predictor (aOR 6.04, 95% CI 3.08–11.87; *p* < 0.001). Higher CRP (aOR 1.74 per SD, 95% CI 1.36–2.23; *p* < 0.001) and WBC (aOR 1.39 per SD, 95% CI 1.11–1.76; *p* = 0.005), as well as lower platelet count (aOR 0.77 per SD, 95% CI 0.61–0.97; *p* = 0.026), were independently associated with prolonged exposure, whereas maternal bacteriological findings and PROM > 18 h were not (Table 4). Continuous predictors are expressed per 1 SD increase, and maternal microbiology tests not performed were handled using a missing-indicator approach.

### 3.7. The NeoSIR Score for Risk Stratification in Suspected Maternal–Fetal Infection

NeoSIR (Neonatal Sepsis Inflammatory Risk) was explored as a simple bedside indicator combining two routinely available parameters: maternal UTI during pregnancy and neonatal CRP > 0.5 mg/dL at 24 h. Each component contributed one point (range 0–2).

Antibiotic initiation was high across NeoSIR strata: 191/206 (92.7%) for score 0, 187/201 (93.0%) for score 1, and 35/35 (100%) for score 2 (Table 5). The high antibiotic initiation rate even in the NeoSIR = 0 group reflects the selected high-risk cohort and a low threshold for empirical treatment in the absence of consistently available culture confirmation; clinical signs and other laboratory abnormalities frequently prompted therapy despite a normal CRP at 24 h.

The overall association between NeoSIR category and antibiotic initiation was not statistically significant (Chi-squared test, *p* = 0.26), and discrimination for antibiotic initiation was limited (AUC = 0.55). Accordingly, we do not propose NeoSIR for clinical cut-offs or treatment initiation decisions in this dataset; it is presented strictly as hypothesis-generating. A NeoSIR score of 2 acted as a specific rule-in flag (specificity 100%) but had low sensitivity (8.5%).

Overall, this two-item composite was feasible to apply in routine care but showed limited ability to separate lower-risk strata under the prevailing low threshold for empirical treatment. Further refinement and validation against culture-confirmed outcomes are required before NeoSIR can be considered a predictive tool.

Accordingly, NeoSIR is presented here as an exploratory indicator intended to support structured reassessment rather than to replace established sepsis calculators or guideline-based observation pathways.

## 4. Discussion

In this selected cohort of late preterm and term newborns evaluated for suspected maternal–fetal infection (MFI), maternal infectious conditions—particularly UTIs and abnormal vaginal secretions—were common and were associated with early clinical management decisions. The very high rate of empirical antibiotic initiation suggests a low threshold for treatment in the absence of consistently available microbiological confirmation, highlighting the tension between timely therapy and antibiotic stewardship.

Screening for TORCH infections, HIV, syphilis, hepatitis B/C, and tuberculosis was more consistently completed among older mothers, reflecting a trend described in recent European analyses showing higher antenatal engagement among women aged 20–34 years compared with adolescents or young adults. Younger mothers, particularly adolescents, exhibited the lowest compliance, increasing the likelihood of undiagnosed infections or missed opportunities for timely treatment [19,20].

Given that COVID-19 during pregnancy may alter inflammatory pathways and predispose to gestational complications), evaluating maternal SARS-CoV-2 history was considered part of the maternal infection assessment [21,22].

Maternal UTIs were strongly associated with neonatal antibiotic therapy initiation and NICU admission in our cohort. One explanation may be that maternal inflammatory states, also described in conditions such as preeclampsia, influence neonatal vulnerability [23].

Recent studies have reaffirmed the relevance of maternal genitourinary infections as predictors of neonatal infectious morbidity, even in late-preterm and term populations [15]. Despite the relatively limited microbiological confirmation available, antibiotic exposure remained high because the cohort was selected as clinically high-risk and local practice maintained a low threshold for empirical treatment under diagnostic uncertainty. Similar observations have been reported in recent perinatal studies, where clinical decision-making increasingly prioritizes maternal infection history and neonatal biomarkers over isolated maternal colonization [24,25,26].

The frequent absence of microbiological confirmation in our population does not exclude clinically relevant inflammatory risk, as antenatal low-grade fetal inflammatory responses have been shown to increase susceptibility to EOS even when intrauterine infection is not microbiologically detectable [27].

The pattern of neonatal outcomes in this cohort reflects the broader challenges faced in secondary-care environments with limited access to blood cultures. Elevated C-reactive protein (CRP) at 24 h of life and NICU admission were strongly associated with prolonged antibiotic therapy, consistent with recent findings that CRP trajectories and clinical instability remain influential predictors of neonatal infection severity when microbiological confirmation is lacking [27,28,29].

Our data also showed that leukocytosis and high neutrophil counts contributed to the decision to initiate antibiotic therapy. Although these hematological markers are not highly specific, they remain widely used as adjunctive tools, particularly in settings where access to more advanced biomarkers (e.g., procalcitonin or presepsin) is limited [30,31,32].

However, the degree of prenatal monitoring did not statistically influence antibiotic initiation or treatment duration once neonatal laboratory abnormalities and clinical symptoms were considered. This pattern is consistent with recent reports that emphasize the dominant role of postnatal indicators in shaping real-time therapeutic decisions in resource-limited settings [15,33].

These findings illustrate the persistent trade-off between ensuring early treatment and avoiding unnecessary antibiotic exposure. Notably, empirical antibiotics were initiated in 306/335 (91.3%) newborns with normal CRP at 24 h (<0.5 mg/dL), underscoring that clinical and maternal-context factors frequently outweighed early biomarker reassurance in routine practice.

Recent European initiatives emphasize minimizing empiric antibiotic use in well-appearing infants with isolated risk factors, primarily through structured observation pathways and improved access to maternal intrapartum data [25,26]. Translating these recommendations to secondary-care settings like ours requires the availability of dependable maternal screening and consistent neonatal monitoring, conditions that remain variable in many parts of Eastern Europe [34].

The NeoSIR indicator was proposed to address the reality that comprehensive risk calculators cannot always be applied when key intrapartum data are missing or prenatal screening is incomplete. NeoSIR represents a simplified exploratory indicator derived from routinely available information; however, its limited discriminatory performance in this cohort underscores the need for cautious interpretation and external validation.

While more advanced tools such as the Kaiser Permanente EOS calculator have demonstrated the ability to reduce unnecessary antibiotic use, their applicability in similar settings is limited, with recent studies underscoring the value of adapted risk scores in environments where access to culture-based diagnostics is limited [16,33].

However, in this cohort—characterized by near-universal empirical treatment—NeoSIR showed limited discrimination for antibiotic initiation (AUC = 0.55) and did not materially separate lower-risk strata. NeoSIR should therefore be viewed as exploratory, and further refinement and external validation against culture-confirmed outcomes are required before considering any clinical decision support role. The selection of NeoSIR components reflects pragmatic availability and early clinical relevance in our setting rather than an attempt to build a comprehensive laboratory-based diagnostic score.

Because antibiotic duration and NICU admission reflect clinical severity and physician judgment, the observed associations are susceptible to confounding by indication: infants who appeared clinically sicker were more likely to be admitted to NICU and to receive prolonged or escalated therapy. Therefore, our findings should be interpreted as describing management-linked associations rather than causal effects.

This study has several limitations. First, blood cultures and other microbiological reference standards were not consistently captured, preventing classification of culture-confirmed early-onset sepsis and limiting inference about diagnostic accuracy. Second, the cohort represents a selected high-risk population rather than an unselected birth cohort, which restricts generalizability and inflates the baseline probability of empirical treatment. This context likely compressed between-group differences and reduced discrimination metrics, including NeoSIR performance, which may not reflect its behavior in more heterogeneous populations. Third, as an observational single-center study, the analyses are vulnerable to confounding by indication and by unmeasured intrapartum variables (e.g., timing of prophylaxis), and we did not fit multivariable predictive models. Finally, outcomes beyond the early hospitalization period were not assessed. In addition, CRP was analyzed using a clinically used threshold because continuous values were not uniformly recorded, limiting assessment of dose–response patterns.

An additional limitation is the restricted ability to classify true neonatal infection using objective confirmation, because microbiological testing was not consistently available and advanced diagnostic modalities (beyond routine cultures) were not systematically performed. Where complete-case modeling was feasible (e.g., the escalation models reported in Table S1), estimates were directionally consistent, supporting the robustness of the main interpretation; however, systematic restriction to tested-only dyads could introduce selection bias in this setting.

The findings highlight the importance of improving prenatal care, particularly universal screening for maternal infections and consistent management of maternal conditions. Increasing antenatal compliance among younger mothers may reduce undiagnosed infections and improve neonatal outcomes [34].

Evidence linking neonatal infection and inflammatory exposure to long-term neurodevelopmental outcomes further supports a cautious approach to early neonatal illness, where clinical and inflammatory indicators may justify empirical treatment despite negative cultures when maternal risk factors are present [24,35].

In the neonatal period, structured risk assessment using simple, widely available indicators could help clinicians balance timely treatment with the principles of antibiotic stewardship. Recent studies show that even modest improvements in risk stratification can significantly reduce unnecessary antibiotic exposure without compromising safety [25,29,36].

Adapting neonatal infection management strategies to the realities of low-resource hospitals will require both improved maternal screening and updated neonatal observation protocols. The approach tested in this study may represent a feasible step toward more consistent and targeted antibiotic use, especially in settings where limited staffing and high patient turnover constrain the practicality of prolonged structured clinical observation [37].

International guidance increasingly supports probability-based management and serial clinical examinations to reduce unnecessary antibiotic exposure in well-appearing infants, while still ensuring prompt treatment for those with clinical deterioration or high risk. Examples include risk calculators and national probability-based protocols, as well as WHO recommendations tailored to resource-limited settings. Adapting these approaches locally will require more consistent capture of intrapartum data and improved access to microbiology to enable timely discontinuation when infection is unlikely [26,29,31,38,39,40,41].

## 5. Conclusions

In a secondary-care setting with limited microbiological resources, the evaluation of suspected maternal–fetal infection relies mainly on maternal history, basic neonatal inflammatory markers, and early clinical signs. Maternal urinary tract infections and CRP elevation at 24 h of life were the most significant factors guiding antibiotic initiation, while leukocytosis, neutrophilia, and NICU admission influenced treatment duration. These patterns reflect the realities of neonatal care in many countries, where incomplete prenatal screening and restricted access to neonatal cultures remain inconsistently available.

Our exploratory NeoSIR indicator may help structure documentation and prompt reassessment in settings where comprehensive sepsis calculators are difficult to apply; however, it showed limited discrimination for antibiotic initiation in this high-treatment cohort and should not be interpreted as a diagnostic or treatment-guiding tool. Multicenter studies incorporating more consistent microbiological confirmation and standardized reassessment protocols are required before any simplified indicator could be considered for clinical decision support.

## Figures and Tables

**Table 1:** Associations between maternal and neonatal infectious status, laboratory findings and antibiotics initiation.

Association	*p*
WBC vs. Gastric culture	0.26 *
RBC vs. Gastric culture	0.23 *
CRP vs. Gastric culture	0.18 *
Categorical CRP vs. Antibiotics	0.032 **
Categorical CRP vs. NICU	0.29 **
Maternal UTI vs. Antibiotics	0.005 **
Cervical culture vs. Antibiotics	0.95 **
Vaginal secretion on admission vs. Antibiotics	0.99 **
Pregnancy monitoring vs. Antibiotics	0.97 **

* Kruskal–Wallis test; ** Chi-square test. Bolded values indicate statistical significance at α = 0.05.

**Table 2:** CRP and antibiotic type.

CRP at 24 h	*n*	Ampicillin	Cefoperazone/Sulbactam	Cefotaxime	Ceftriaxone	Ciprofloxacin	Imipenem/Cilastatin	Meropenem	No Antibiotics
Normal (<0.5 mg/dL)	335	281 (83.9%)	2 (0.6%)	1 (0.3%)	4 (1.2%)	3 (0.9%)	2 (0.6%)	13 (3.9%)	29 (8.7%)
Elevated (≥0.5 mg/dL)	107	102 (95.3%)	0 (0.0%)	0 (0.0%)	3 (2.8%)	0 (0.0%)	0 (0.0%)	2 (1.9%)	0 (0.0%)

Data are presented as *n* (%). CRP refers to the value measured at ~24 h of life. Broad-spectrum agents (e.g., ceftriaxone, imipenem–cilastatin, meropenem) reflect selective escalation in a minority of cases and were not used as routine first-line therapy.

**Table 3:** Maternal infection screening indicators and outcomes (univariable associations).

Maternal Variable	Outcome	*p* Value
Maternal UTI	Antibiotic initiation	**0.005**
Vaginal secretions during hospitalization	Antibiotic initiation	0.99
Cervical culture status	Antibiotic initiation	0.95
Maternal UTI	NICU admission	0.91
Vaginal secretions during hospitalization	NICU admission	0.93
Cervical culture status	NICU admission	0.97

Data are presented as *p* values from Chi-square tests; *p* values are descriptive. Bolded values indicate statistical significance at α = 0.05.

**Table 4:** Multivariable predictors of prolonged antibiotic exposure (≥10 antibiotic-days).

Predictor	Adjusted OR (95% CI)	*p*-Value
NICU admission (yes vs. no)	6.04 (3.08–11.87)	<0.001
Neonatal CRP at 24 h (per SD)	1.74 (1.36–2.23)	<0.001
Neonatal WBC at 24 h (per SD)	1.39 (1.11–1.76)	0.005
Neonatal platelets at 24 h (per SD)	0.77 (0.61–0.97)	0.026
Gestational age (per SD)	1.05 (0.78–1.43)	0.732
Birth weight (per SD)	0.91 (0.70–1.19)	0.492
Male sex (vs. female)	0.88 (0.55–1.40)	0.582
PROM > 18 h (yes vs. no)	1.78 (0.40–7.99)	0.451
Maternal UTI during pregnancy (yes vs. no)	1.28 (0.76–2.15)	0.348
Positive cervical culture (yes vs. no)	1.34 (0.79–2.26)	0.282
Neonatal resuscitation at birth (yes vs. no)	0.83 (0.51–1.37)	0.470
EOS risk at birth (log, per SD)	1.11 (0.84–1.47)	0.470

Adjusted odds ratios (aORs) from logistic regression; outcome defined as ≥10 antibiotic-days. Continuous predictors are expressed per 1 SD increase; EOS risk at birth was log-transformed. Maternal microbiology tests not performed were handled using a missing-indicator approach.

**Table 5:** NeoSIR score and antibiotic therapy.

Score NeoSIR	Antibiotherapy (*n*, %)	
	YES	NO
0	191 (92.7%)	15 (7.3%)
1	187 (93.0%)	14 (7.0%)
2	35 (100.0%)	0 (0.0%)

## Data Availability

Data supporting reported results can be found at anca.vulcanescu@umfcv.ro, mirela.siminel@umfcv.ro; due to the need to uphold intellectual property rights and confidentiality agreements, ensure the integrity and accuracy of ongoing analyses, and comply with ethical and regulatory guidelines that govern data dissemination before formal publication.

## Supplementary Materials

The following supporting information can be downloaded at: https://www.mdpi.com/article/10.3390/biomedicines14030538/s1, Table S1. Multivariable logistic regression models for broad-spectrum escalation (expanded coverage) among antibiotic-treated eligible newborns (complete-case *n* = 380; events = 77).
