# Supplementary material for: Maternal–Fetal Infectious Risk and Early Antibiotic Treatment Patterns in Late Preterm and Term Newborns in a Romanian Secondary-Care Maternity: A Six-Month Observational Cohort
Source: Biomedicines. 2026 Feb 27;14(3):538. doi: 10.3390/biomedicines14030538 (PMC13024130; doi:10.3390/biomedicines14030538)
Supplement: Supplementary file 1 [file biomedicines-14-00538-s001.zip › biomedicines-4147129-supplementary.pdf]

Table S1. Multivariable logistic regression models for broad-spectrum escalation (expanded coverage) among antibiotic-treated eligible newborns (complete-case n = 380; events = 77).

Covariate	Model 1 aOR (95% CI)	p	Model 2 aOR (95% CI)	p
Gestational age (per 1 SD)	0.86 (0.66-1.13)	0.274	0.97 (0.74-1.27)	0.821
Rupture of membranes >18 h (yes vs no)	1.59 (0.40-6.33)	0.508	1.10 (0.26-4.62)	0.901
Neonatal resuscitation at birth (yes vs no)	0.93 (0.53-1.64)	0.813	0.63 (0.33-1.18)	0.147
CRP at presentation (per 1 SD)	1.03 (0.79-1.34)	0.849	1.00 (0.76-1.30)	0.977
WBC at presentation (per 1 SD)	0.98 (0.77-1.26)	0.902	1.05 (0.83-1.33)	0.698
Platelets at presentation (per 1 SD)	0.78 (0.61-1.01)	0.060	0.87 (0.67-1.13)	0.287
NICU admission (yes vs no)	—	—	5.33 (2.70-10.52)	<0.001

Adjusted odds ratios (aOR) with 95% confidence intervals (CI) from logistic regression. Model 1 included gestational age, rupture of membranes >18 h, neonatal resuscitation at birth, and baseline laboratory markers (CRP1, WBC1, PLT1) (pre-specified a priori; no stepwise selection). Model 2 additionally adjusted for NICU admission. Continuous predictors are reported per 1 SD increase. Events-per-variable were approximately 12.8 (Model 1) and 11.0 (Model 2). Broad-spectrum escalation (expanded coverage) was defined as exposure to any third-generation cephalosporin, carbapenem, fluoroquinolone, colistin, linezolid, or metronidazole during hospitalization.
